# Evaluating Operational Specifications of Point-of-Care Diagnostic Tests: A Standardized Scorecard

**DOI:** 10.1371/journal.pone.0047459

**Published:** 2012-10-31

**Authors:** Jonathan D. Lehe, Nádia E. Sitoe, Ocean Tobaiwa, Osvaldo Loquiha, Jorge I. Quevedo, Trevor F. Peter, Ilesh V. Jani

**Affiliations:** 1 Clinton Health Access Initiative, Maputo, Mozambique; 2 Instituto Nacional de Saúde, Maputo, Mozambique; 3 Department of Mathematics and Informatics, Universidade Eduardo Mondlane, Maputo, Mozambique; University of Cape Town, South Africa

## Abstract

The expansion of HIV antiretroviral therapy into decentralized rural settings will increasingly require simple point-of-care (POC) diagnostic tests that can be used without laboratory infrastructure and technical skills. New POC test devices are becoming available but decisions around which technologies to deploy may be biased without systematic assessment of their suitability for decentralized healthcare settings. To address this, we developed a standardized, quantitative scorecard tool to objectively evaluate the operational characteristics of POC diagnostic devices. The tool scores devices on a scale of 1–5 across 30 weighted characteristics such as ease of use, quality control, electrical requirements, shelf life, portability, cost and service, and provides a cumulative score that ranks products against a set of ideal POC characteristics. The scorecard was tested on 19 devices for POC CD4 T-lymphocyte cell counting, clinical chemistry or hematology testing. Single and multi-parameter devices were assessed in each of test categories. The scores across all devices ranged from 2.78 to 4.40 out of 5. The tool effectively ranked devices within each category (p<0.01) except the CD4 and multi-parameter hematology products. The tool also enabled comparison of different characteristics between products. Agreement across the four scorers for each product was high (intra-class correlation >0.80; p<0.001). Use of this tool enables the systematic evaluation of diagnostic tests to facilitate product selection and investment in appropriate technology. It is particularly relevant for countries and testing programs considering the adoption of new POC diagnostic tests.

## Introduction

Decentralization of HIV antiretroviral treatment (ART) in resource-limited settings will require increased access to simple diagnostic technologies such as point-of-care (POC) tests suitable for use in primary health care facilities with minimal infrastructure, resources and skills. UNAIDS has identified POC tests as a key pillar of its HIV Treatment 2.0 framework in resource-limited settings [1]. While few HIV POC tests other than rapid serological test strips are widely used, global HIV testing demands are likely to be increasingly met by POC technologies for CD4+ T-cell counting (CD4), viral load quantification (VL), early infant diagnosis (EID), and HIV drug toxicity monitoring and resistance testing [2]–[4]. Point-of-care tests have the potential to improve access to ART and treatment program efficiency; for example recent studies demonstrated that POC CD4 testing reduced patient loss-to-follow-up and increased enrollment in ART in primary healthcare settings [5]. Given the growing uncertainty in global health funding [6], [7], interventions to improve ART efficiency are needed.

In recent years, investment by development donors and industry in the development of POC technologies for CD4, viral load and EID has resulted in a broader pipeline of products expected to reach the market in the next 1–3 years [8], [9]. In addition, a number of products for POC drug toxicity monitoring (clinical chemistry and hematology) exist but are not used widely. As ART is decentralized further, countries and testing programs will increasingly make decisions on which technologies to implement. Deployment of new technology on a national scale requires significant investment and diagnostic devices remain in use for years, hence selection of appropriate technology is important. However, the identification of appropriate products can be hampered by the lack of adequate and impartial information on their operational characteristics and suitability for the settings in which they will be used. The selection process may be non-standardized, subjective and susceptible to bias. Critical features defining the utility of each device in the targeted setting, e.g. test throughput, heat-stability, quality control, availability of service support, portability, ease of use or ability to run on batteries may be overlooked or assessed inconsistently across different products.

Here we describe the design of an objective, quantitative scorecard to assess the operational specifications POC test devices and its application to products for POC HIV CD4 count and drug toxicity monitoring.

## Methods

### Pre-selection of products

To identify products for assessment, a survey of CD4, hematology, and clinical chemistry products labeled as POC and available on the market was conducted. The following definition of POC was used: a fully or partially automated table-top, portable, handheld or disposable device able to be operated in a non-laboratory environment by non-technical staff to deliver a same-day, on-site, clinically relevant, diagnostic test result. A survey to identify POC diagnostic technologies to assess with the tool was undertaken. Products were identified from lists of known technologies maintained by the Instituto Nacional de Saúde, internet searches, and company enquiries. Search terms included “Point-of-care, test, diagnostics, technology”. All products identified in this search were catalogued and then assessed if commercially available by contacting the suppliers. All products that were not commercially available were excluded from the study. Information on product specifications was provided by suppliers via brochures and formal communications. Products were then pre-screened for their ability to conduct one or more of the following target tests, CD4 T+ lymphocyte enumeration, liver function (ALT and AST), kidney function (creatinine), glucose, cholesterol, full or partial blood cell count, and hemoglobin.

The shortlisted products were separated into five categories: (i) CD4+ T-cell count analyzers; (ii) multiple-parameter clinical chemistry analyzers (conducts more than 3 different types of tests); (iii) limited-parameter clinical chemistry analyzers (conducts 1–3 different tests); (iv) multiple-parameter hematology analyzers and; (v) limited-parameter hematology analyzers.

### Scorecard Design

The selected devices were subjected to a quantitative analysis using a scorecard that rated each product based on a pre-defined set of ideal POC specifications, including factors such as portability, power source, heat stability, daily throughput, access to service and maintenance, availability of quality controls, ease of use, and cost. Thirty assessment criteria were scored for each product across six major categories as follows:

*POC Features of Equipment* - the degree to which a product's equipment can be deployed in settings with limited infrastructure, e.g. portability, throughput, and power source.

*POC Features of Consumables* - the degree to which the product consumables were appropriate for use in settings with limited infrastructure, e.g. heat stability and shelf life of reagents and controls.

*Ease of Use* - how suitable a product is for operators with minimal technical training, and the simplicity of the testing procedure.

*Quality Control* - assesses whether a product provides sufficient internal quality control mechanisms and is compatible with external quality assurance schemes.

*Cost* - the relative cost the equipment and consumables.

*Distribution and Service* - assesses whether local distribution, technical service and after-sales support is available.

The scorecard allows for each device to be scored on a scale of 1–5 (with 5 representing ideal) to assess the device's utility for POC testing in low-resource, primary health care settings. This overall score was made up from individual scores of the 30 assessment criteria each of which had pre-assigned score levels between 1 and 5 for different specifications. For example, the scoring for electrical requirement was as follows: No external power required: 5 points; rechargeable battery operated: 3 points; requires continuous stable electricity: 0 points. In addition, each individual score was also weighted based on its relative importance compared with other criteria. The scorecard with the criteria, scoring thresholds and weightings is provided in Table 1.

**Table 1:** Diagnostic technology scorecard assessment criteria with scoring thresholds and weightings.

Criteria	Scoring Thresholds	Weighting	Type of Criteria	Key POC Criteria Y/N
**POC Features of Equipment**		**29.0%**		
Test Parameters	More parameters relative to the other products in its category = 5, Average number of parameters relative to the other products in its category = 3, Fewer parameters relative to the other products in its category = 1	4.0%	Objective	N
Type of Technology Presentation	Disposable = 5, Handheld = 3, Tabletop = 1	3.0%	Objective	N
Portability	Could Be Transported By Hand = 5, Could Be Transported By Vehicle - 3, Could Not Be Transported = 1	3.0%	Subjective	N
Throughput	50+ = 5, 25–49 = 3, 1–24 = 1	3.0%	Objective	N
Power Source	No Power Required, or Battery-Powered = 5, Requires Electricity = 1	3.0%	Objective	Y
Alternate Power Sources	Alternate Power Source Available with actual products available, such as solar, plug in to car cigarette lighter, etc. = 5, No Alternate Power Source Available = 1	1.0%	Objective	N
External Equipment	Not Required = 5, Required = 1	2.0%	Objective	N
Batching	Possible = 5, Not Possible = 1	3.0%	Objective	N
Result Display	Printed Results & Displayed On Device (if device-based), or Disposable Test = 5, Results Displayed On Device Only (if device-based) = 1	2.0%	Objective	N
Result Storage	Results Stored & Interface (if device-based), or Disposable Test = 5, Results Stored but No Interface (if device-based) = 3, Results Not Stored (if device-based) = 1	2.0%	Objective	N
Instrument Connectivity	Capability for the instrument to communicate (e.g. wireless) data to outside location = 5, No communication capability = 1	2.0%	Objective	N
Installation	Vendor Technician Not Required = 5, Vendor Technician Required = 1	1.0%	Subjective	N
**POC Features of Test Consumables**		**8.0%**		
Heat Stability of Reagents and Controls	Max 40 degrees for 3 months = 5, Max 25–40 degrees for 3 months = 3, Max <25 degrees for 3 months = 1	5.0%	Objective	Y
Type of Sample Collection Tubes Required	Uses standard tubes or no tubes = 5, Requires specialized tubes = 1	1.0%	Objective	N
Shelf Life	>12 Months = 5, 6–12 Months = 3, <6 Months = 1	2.0%	Objective	N
**Ease of Use**		**34.0%**		
Operator Skills	Layperson = 5, Semi-Skilled Technician = 3, Highly Skilled Technician = 1	5.0%	Subjective	Y
Routine Maintenance	No Routine Maintenance = 5, Routine Maintenance By Operator = 3, Routine Maintenance By Technician = 1	3.0%	Subjective	N
Reagent & Control Preparation	Not Required = 5, Minimal Preparation Required = 3, Significant Preparation Required = 1	5.0%	Subjective	Y
Daily Calibration	Auto-Calibration or Calibration not required = 5, Manual Calibration = 1	3.0%	Objective	N
Steps in Sample Preparation and Testing	Few Easy Steps and No Sample Preparation Required = 5, Few Easy Steps but One Step Requiring Pipette or Capillary Tube = 4, 2–4 Easy Steps e.g. Additional Pipetting, Incubation, etc. = 3, Additional Sample Preparation = 2, Complex Technical Steps = 1	5.0%	Subjective	Y
Type of Sample	Capillary Blood = 5, Venous Blood, Serum or Plasma = 1	5.0%	Objective	N
Precise Sample Measurement	No manual pipetting of precise quantity required = 5, Manual pipetting of precise quantity required = 1	5.0%	Objective	N
Waste	Minimal Solid Waste = 5, Minimal Liquid and Solid Waste = 3, Liquid and Solid Waste = 1	3.0%	Subjective	N
**Quality Control**		**10.0%**		
Internal Quality Control	Internal QC Available = 5, No Internal QC Available = 1	5.0%	Objective	Y
External Quality Control (EQA)	Compatible with commercial EQA = 5, Not compatible with commercial EQA = 1	5.0%	Objective	N
**Cost**		**10.0%**		
Capital Cost of Equipment	<$1,000 = 5, $1,000–5,000 = 3, >$5,000 = 1	5.0%	Objective	Y
Reagent, Consumable and Control Cost	<$2 per test AND<$100 per month for controls = 5; $2–10 per test AND/OR $100–200 per month of controls = 3; >$10 per test OR>$200 per month of controls = 1	5.0%	Objective	Y
**Distribution and Service**		**9.0%**		
Corrective Service and Maintenance	Easily Available in-country = 5, Sporadically or regionally available, e.g. only in certain countries or only available outside Africa = 1	3.0%	Subjective	Y
Supply Chain and Distribution	Easily Available in-country = 5, Sporadically or regionally available, e.g. only in certain countries or only available outside Africa = 1	3.0%	Subjective	N
Timing and Regulatory Status	Available and Approved Now = 5, Available <1 Year = 3, Available >1 Year = 1	3.0%	Objective	N
		**100.0%**		

Eight of the 30 criteria were considered key POC characteristics. These included features such as simplicity of use (requiring minimal operator skills), low cost instruments and consumables, presence of internal quality control, battery power, heat stability, availability of service and maintenance (Table 1).

### Technology Assessments

Each of the products was scored by four independent scorers that were blinded to each other's assessments. The scorers were laboratory technicians with training and experience in medical diagnostics in low-resource settings. The overall score for each device was determined as the average of the four scores, and an unbiased arbitrator collated and analyzed the score data, and resolved any clear errors or discrepancies. Most criteria were objective (Table 1) and therefore if discrepancies across scorers arose related to these criteria, the scores were reviewed with the scorers to determine whether an error may have occurred. Any scores provided in clear error were corrected. Certain other criteria were more subjective and open to scorer interpretation (Table 1). If discrepancies across scorers arose related to these subjective criteria, they were investigated to ensure that there were no obvious errors or inconsistencies, but otherwise these discrepancies were assumed to be valid.

### Statistical Analysis

Statistical analyses were done to evaluate the rank of each product and the reliability of the tool for each class of technology. As all products were judged by the same scorers, the Friedman test was used for a one-way repeated measures analysis of variance for ranks evaluation. We performed a two-way mixed model assuming absolute agreement and used single measures inter-class correlation (ICC) to assess the inter-rater reliability.

These analyses were conducted using SAS version 9.2 (SAS Institute Inc. USA). Tests used 2-sided p-values with α = 0.05 for level of significance and 95% confidence intervals.

### Ethics Statement

This study was reviewed and approved by the Mozambique National Health Bioethics Committee. No human subjects participated in this study and no patient samples or patient data were used.

## Results

Of 49 diagnostic devices identified by the survey that met our definition of POC, 19 performed one or more of the target test parameters (Table 2). The remaining products did not conduct any of the target parameters and were not evaluated using the scorecard, e.g. blood gas analyzers were not included in the study. The 19 selected devices consisted of 2 CD4 analyzers, 6 multi-parameter blood chemistry analyzers, 4 limited-parameter chemistry analyzers, 3 multi-parameter hematology analyzers, and 4 hemoglobin analyzers (limited parameter hematology instruments).

**Table 2:** Point-of-care medical diagnostic products evaluated with the scorecard.

Technology Category	Product
CD4+ T-cell count analyzers	Alere Pima
	PointCare NOW
Multiple-parameter clinical chemistry analyzers	Abbott i-STAT
	Cholestech LDX
	Piccolo Xpress
	Roche Reflotron Plus
	Spotchem EZ
	Vitros DT
**Limited-parameter clinical chemistry analyzers**	
	AVL 9180
	Combur-Test (Urisys Reader)
	Nova StatSensor Creatinine Meter
	Roche Accutrend Plus
**Multiple-parameter hematology analyzers**	
	PointCare NOW
	QBC Star
	Sysmex pocH-100i
**Limited-parameter hematology analyzers**	
	HemoCue HB201+
	HemoPoint H2
	ITC HgB Pro
	Stat-Site M

The scores across all product categories ranged from 2.78 to 4.40 out of 5 (Table 3). The range of scores enabled the ranking the devices. The ranking was statistically significant (p<0.01) in all product categories except for the CD4 and multi-parameter hematology devices (Table 3). Amongst the set of products evaluated in this study, the lowest ranking products achieved scores within 15–30% of the highest score of each category, suggesting relative homogeneity of operational characteristics as assessed by this tool. The widest range of scores by technology was seen amongst the multiple-parameter chemistry instruments indicating that these products were more diverse in characteristics, while scores amongst the hemoglobin meters were closest indicating similar characteristics in this product category (Table 3).

**Table 3:** Score, rank and inter-class correlation of point-of-care technologies assessed with the scorecard.

Analyzer Category	Product	Average Score (Range)	Mean Rank	p-value	Percent of Top Score in Category	Intra-class correlation*
CD4 T+ cell count	1	4.00 (3.83–4.08)	1	p = 0.125	100.0%	0.862 (0.777–0.924)
	2	3.06 (2.82–3.16)	2		76.4%	0.845 (0.755–0.916)
Multiple-parameter clinical chemistry	1	3.89 (3.79–4.04)	1	p = 0.01	100.0%	0.865 (0.782–0.926)
	2	3.56 (3.46–3.66)	2		91.4%	0.927 (0.879–0.961)
	3	3.33 (3.29–3.43)	3.25		85.7%	0.852 (0.762–0.918)
	4	3.26 (3.14–3.44)	3.75		83.8%	0.873 (0.794–0.931)
	5	3.07 (2.99–3.23)	5		78.8%	0.921 (0.868–0.958)
	6	2.78 (2.65–2.88)	6		71.4%	0.959 (0.931–0.978)
Limited-parameter clinical chemistry	1	4.15 (3.98–4.40)	1	p = 0.001	100.0%	0.864 (0.776–0.926)
	2	4.10 (3.96–4.30)	2		98.8%	0.881 (0.805–0.935)
	3	3.89 (3.76–3.96)	3.25		93.7%	0.955 (0.924–0.976)
	4	3.39 (3.18–3.76)	3.75		81.8%	0.834 (0.735–0.908)
Multiple-parameter hematology	1	3.75 (3.56–3.94)	1	p = 0.069	100.0%	0.855 (0.767–0.920)
	2	3.73 (3.58–3.84)	2.5		99.5%	0.979 (0.683–0.886)
	3	2.96 (2.66–3.12)	2.5		79.1%	0.837 (0.738–0.909)
Limited-parameter hematology	1	4.17 (4.01–4.40)	1	p = 0.001	100.0%	0.974 (0.955–0.986)
	2	3.75 (3.61–3.88)	2		90.0%	0.905 (0.844–0.949)
	3	3.62 (3.55–3.70)	1.25		86.8%	0.972 (0.952–0.985)
	4	3.57 (3.46–3.64)	1.75		85.6%	0.802 (0.688–0.888)

* all p-values<0.0001.

Of the 30 scorecard criteria, eight were considered key POC features that might disqualify devices from use. When assessing the 19 products for these key POC features, mean scores were high (>4.0) for the following criteria: availability of battery power, ease of reagent preparation and simplicity of test procedure, and availability of internal quality control. Mean scores were low (<3.0) for the following criteria: cost of equipment and reagents, heat stability of reagents (primarily due to low stability of control reagents) and weak distribution and service (due to lack of in-country providers). The multi-parameter chemistry, hematology and CD4 platforms scored lower on many of the key POC parameters than the single parameter platforms.

There was a high degree of agreement in the scores given to each product across the four scorers as well as in the scores for each of the 30 assessment criteria, with intra-class correlations (ICC) greater than 0.80 (p<0.001) for each product (Table 3), demonstrating the internal consistency of the tool.

**Figure 1:**
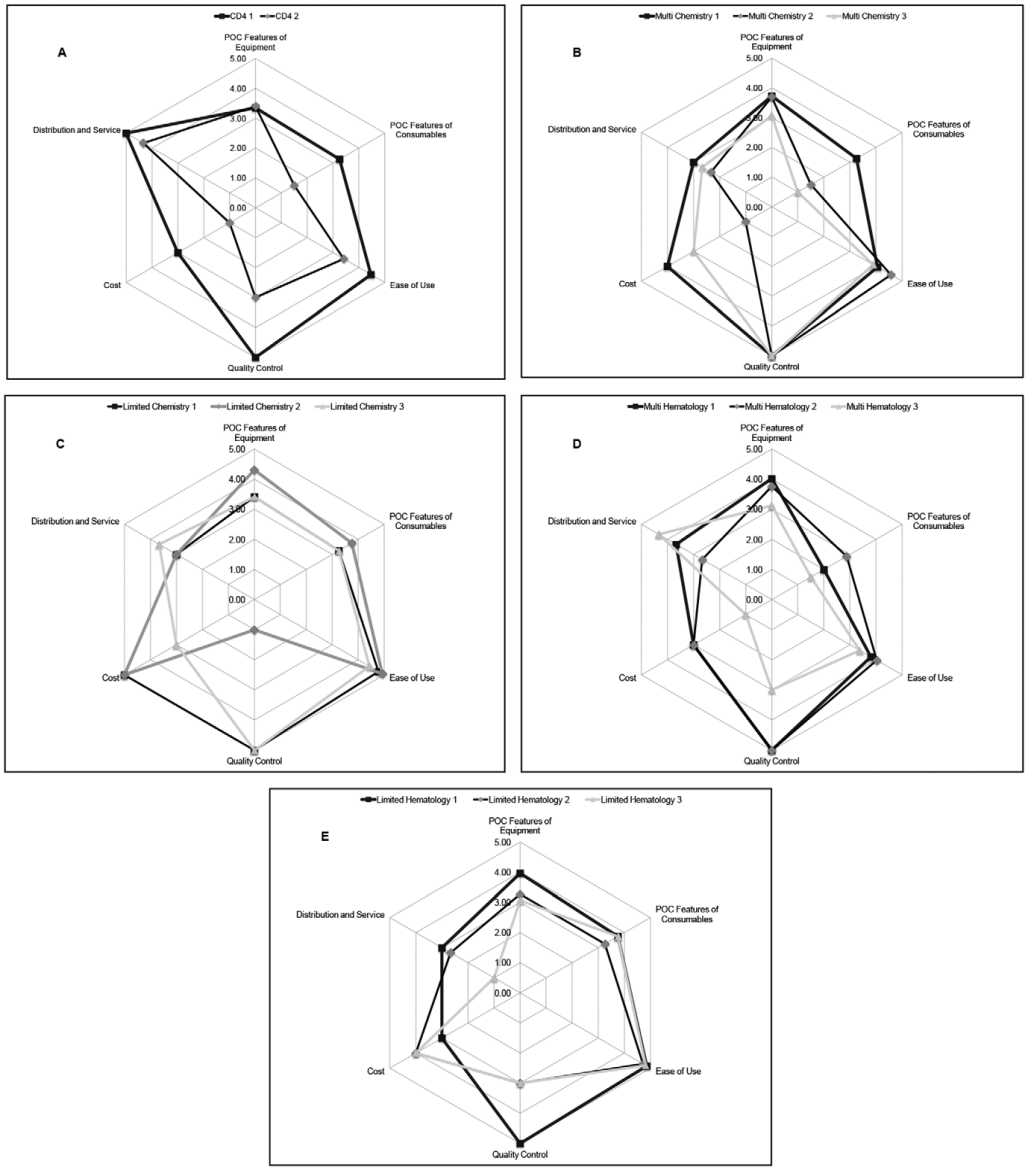
Spider-plot scores of the highest performing technologies.

A simplified representation of the performance of the top 2–3 scoring devices in each product category for technologies is illustrated in Figure 1. These spider plots chart the combined scores within the six major categories of assessment: cost, POC characteristics of the instrument, POC characteristics of the consumables, distribution and service, quality control and ease of use. These plots allow rapid assessment of performance across major operational characteristics and demonstrate that no product scored high in all areas.

## Discussion

We developed and tested an objective and standardized non-laboratory methodology for evaluating POC diagnostic devices being considered for deployment in decentralized healthcare settings. This tool enables the selection of products using unbiased criteria based on a set of ideal POC specifications that take into account key operational features such as portability, battery use, heat stability and ease of use, which are required for tests conducted in rural and low-resource settings with minimal infrastructure, electricity and skills. When applied to 19 currently available diagnostic devices, the scorecard successfully ranked the products and systematically identified the strengths and weaknesses of each platform.

The assessment method described here may reduce opportunity for bias in product selection and help health care programs invest in appropriate technology. The scorecard provides a way to objectively navigate the range of information provided by vendors and other sources, a task complicated by the large number of commercially available test devices and inconsistent information available for different products. While this scorecard does not replace assessment of clinical performance on patient samples, it can be used to shortlist devices for clinical evaluation or can be combined with test performance data to guide adoption and deployment decisions.

Although the scorecard ranked products, many scores were close and performance of products in different operational areas cannot be understood from the overall scores. The spider plots enabled key characteristics of different products to be more easily compared, as illustrated in Figure 1. These plots allow quick assessment of the trade-offs between technologies in areas such as quality control, ease of use, cost and service availability, especially amongst closely ranked products with similar overall scores.

Of the products evaluated, the multi-parameter chemistry, hematology and CD4 platforms generally scored lower than the single parameter CD4 platforms, indicating that the latter devices had more ideal POC features. As single parameter devices have drawbacks associated with the need to maintain multiple different platforms, future technology development should focus on improving the POC features of multi-parameter platforms to enable their deployability in resource-limited settings. Furthermore, across the 19 products, certain key POC features generally scored high, such as quality control and ease of use, while other characteristics such as cost and availability of service and distribution tended to score low. This highlights areas for manufactures and distributors to focus on when considering ways to improve design and delivery of current and future products.

The weighting and scoring system of the tool was based on the set of pre-defined ideal POC characteristics. These weights and scores can be changed if needed to customize the tool for different settings. In addition, a simplified version of the tool may be applicable in certain settings, for example based on the eight key POC criteria. The tool does not provide an absolute cut-off to define high performing or low performing devices, as this may vary with each set of products analyzed. Instead, within each category the tool ranks devices relative to each other in order to prioritize them for further evaluation and/or implementation, or to identify tradeoffs that need to be considered. For example, if ease of use, cost and service availability or good quality control are priorities for a testing program, devices that score higher on these parameters can be short-listed over products that have other strengths.

An advantage of the scorecard approach is that both qualitative and quantitative technology characteristics can be assessed. It therefore complements laboratory or clinical evaluations, which typically assess quantitative characteristics such as sensitivity and specificity, predictive values, bias, precision and reproducibility [10], [11]. One drawback of the scorecard is the potential for inaccurate scores due to incorrect information on products. As some of the data used to populate the tool may not be directly verifiable without a sample instrument, inaccurate information can influence the results. It is therefore necessary to confirm the source and integrity of the information used. Another drawback is that some information may not be known for new products that have had limited use, such as whether the technology will be compatible with external quality assurance schemes. In addition, 10 of the 30 assessment criteria scored were subjective and potentially open to different interpretation. These 10 criteria accounted for 35% of the total score weightings for each device. However, the results of the inter-rater reliability analysis in this study suggest that the overall variation in product scores was predominantly due to actual differences in product characteristics, rather than the subjectivity of certain assessment criteria or random variation attributed to the scorers.

The scorecard described here for assessing the operational characteristics of POC products can help improve the systemic validation of diagnostic tests, an area which is currently deficient [12]–[14]. Laboratories and public health managers faced with the challenge of selecting appropriate POC diagnostic devices can use this tool to help guide investment in new technologies and their deployment. This is particularly relevant because a number of new POC technologies are becoming available at a time when funding to expand treatment programs for HIV and other diseases is less certain. Careful selection and deployment of technologies that have both good technical performance and the appropriate operational features will be required.
